# The Role of *Brassica* Bioactives on Human Health: Are We Studying It the Right Way?

**DOI:** 10.3390/molecules25071591

**Published:** 2020-03-30

**Authors:** Sarai Quirante-Moya, Paula García-Ibañez, Francisco Quirante-Moya, Débora Villaño, Diego A. Moreno

**Affiliations:** 1 Centro de Salud Callosa del Segura, Paseo Enrique Tierno Galvan, 19, E-03360 Alicante, Spain; quirante_sar@gva.es; 2 CEBAS-CSIC, Department of Plant Nutrition. Aquaporins Group, Campus Universitario de Espinardo-25, E-30100 Murcia, Spain; pgibanez@cebas.csic.es (P.G.-I.); franciscojose.quirantem@um.es (F.Q.-M.); 3 CEBAS-CSIC, Department of Food Science and Technology, Phytochemistry and Healthy Foods Lab, Research Group on Quality, Safety and Bioactivity of Plant Foods, Campus de Espinardo-25, E-30100 Espinardo, Murcia, Spain; 4 Faculty of Health Sciences, Department of Pharmacy, Universidad Católica de San Antonio de Murcia (UCAM), Campus de los Jerónimos, E-30107 Guadalupe, Murcia, Spain; dvillano@ucam.edu

**Keywords:** *Brassica*, clinical trials, glucosinolates

## Abstract

*Brassica* vegetables and their components, the glucosinolates, have been suggested as good candidates as dietary coadjutants to improve health in non-communicable diseases (NCDs). Different preclinical and clinical studies have been performed in the last decade; however, some concerns have been posed on the lack of established and standardized protocols. The different concentration of bioactive compounds used, time of intervention or sample size, and the lack of blinding are some factors that may influence the studies’ outcomes. This review aims to analyze the critical points of the studies performed with *Brassica*-related biomolecules and propose some bases for future trials in order to avoid biases.

## 1. Introduction

Nowadays, the incidence of non-communicable diseases (NCDs), including obesity, diabetes, cancer, and other chronic conditions, is increasing and showing high mortality indexes worldwide (https://www.who.int/nmh/topics/es/). These diseases are multifactorial, but it has been described that lifestyle, such as smoking or sedentary life, contributes to the prevalence of the NCDs. As a result, different prevention strategies have been developed, mainly the promotion of healthy habits, recommending avoiding a sedentary life, quitting smoking and the consumption of alcoholic drinks, and keeping a healthy diet [1]. To emphasize the importance of these strategies, it has been proven that these habits have an important role in the prevention of cardiovascular diseases and type-II diabetes [2].

**Figure 1:**
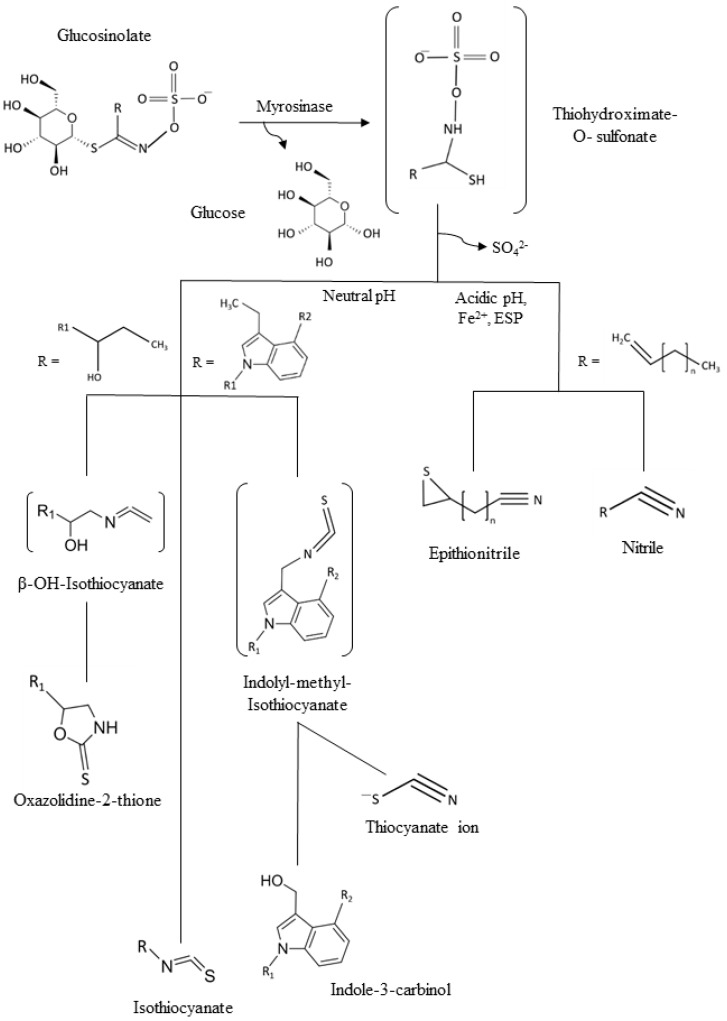
General scheme of the glucosinolates (GSLs) and common hydrolysis products. ESP: Epithiospecifer proteins.

Since one of the main points of intervention for a healthy lifestyle is diet, global health recommendations are always encouraging the message of eating a minimum of five portions of fruits and vegetables daily [3,4]. Vegetables are not only a natural source of amino acids and minerals, but also of phytochemicals [5]. In particular, cruciferous vegetables, like broccoli, cabbage, red radish, or Brussels sprouts, are consumed worldwide not only because of their culinary value being popular in many countries with plates being part of the culture legacy, but also because of their diverse content in phytochemicals with high health-promoting benefits [6,7]. Between them vitamin C, polyphenols, and minerals can be found. However, one of the most relevant biomolecules are glucosinolates (GSLs), mainly found in plant species and varieties of the order Brassicales, mainly Brassicaceae as the most well-known representatives of this horticultural, botanical, and socioeconomically relevant botanical family [8]. They consist of a basic structure of a thiohydroximate-*O*-sulfate group with a glycosylation and, depending on which amino acid they are derived from, a different side chain [8]. GSLs are stable secondary metabolites, but after tissue disruption, they are hydrolyzed by the enzyme myrosinase (EC 3.2.1.147), generating different GSL hydrolysis products (Figure 1) including bioactive isothiocyanates (ITCs) if the pH is 5–8, in the range of neutrality. In some plants, epithiospecifier proteins exist and can modify the outcome of the hydrolysis process, typically to promote other products than ITCs, as it is the case of nitrile specifier protein originating simple nitriles and elemental sulfur [8,9,10]. On the other hand, the epithiospecifier proteins (ESP) with similar function may also carry out a different reaction for the few aliphatic GSLs with a terminal double bond by adding the sulfur to this bond, forming an epitionitrile. The production of thiocyanates is produced at pH > 8, and oxazolidine-2-thiones can be formed if a hydroxyl group is present on carbon 2 (beta position) of the glucosinolate [10,11].

The effect of cooking practices on the content of GSLs and degree of conversion to ITC/nitrile are also important when considering the use of cruciferous foods for clinical studies, and a relevant source for clarification is the review of Nugrahedi et al. [12].

Sulforaphane was discovered by Prochazka [13], but it became widely known after the study by Fahey et al. [14] describing its involvement in cancer prevention. That pioneering study was in some ways misleading, as the protocol used (addition of purified myrosinase enzyme to chemical extracts of broccoli) could have masked the normal tendency of broccoli to form nitriles. Importantly, natural glucoraphanin and sulforaphane both have a chiral sulfinyl group in the side chain [15]; the natural molecule is pure R-isomer. In contrast, some commercial glucoraphanin and sulforaphane has a sulfinyl group that is a mixture of the R and S isomers, because of semisynthetic preparation by chemical oxidation of a more easily available glucosinolate. Importantly, the artificial racemic mixture has different biological properties than the natural single stereoisomer [16,17], which is an important fact to know in the evaluation of published studies.

Diverse bioactivities have been reported for ITCs, being strong inducers of phase II detoxification enzymes [18], their anti-carcinogenic effect [19], or their anti-inflammatory properties [20]. Between aliphatic GSLs, glucoraphanin (GRA) is one of the most studied, since its resultant ITC, sulforaphane (SFN), has shown diverse properties on human health For example, it has been described that SFN exerts its main function acting over the nuclear factor erythroid 2-related factor 2 (Nrf2) [13,14]. In normal conditions, Keap1 represses the interaction between the transcription factor Nrf2 and the sequence motif present in nuclear DNA known as antioxidative response element (ARE) through sequestration and the subsequent ubiquitination and proteasomal degradation of Nrf2 [21]. However, the interaction between SFN and Keap1 interferes with the suppression of Nrf2, allowing the transcription of phase II cytoprotective proteins [22]. Furthermore, also the subject of study has been the intervention of SFN in the nuclear factor kappa-light-chain-enhancer of activated B cells (NFκB), decreasing its capability of binding target sequences related to inflammatory processes, such as tumor necrosis factor (TNF-α) [23].

Another abundant GSL present in cruciferous vegetables is glucobrassicin (GB) [24,25], whose main degradation product is ascorbigen [26]. However, its corresponding indole-3-carbinol (I3C) and its condensation product 3,3-diindolylmethane (DIM) [27] have been the main focus of medical research because they were readily commercially available. Glucobrassicin was discovered as the precursors of the already known ascorbigen [28] I3C and DIM were found to be formed in vitro, but not in vivo. From the very fast formation of ascorbigen at plant pH, it is well established that I3C is neither an intermediate in the in vivo formation of ascorbigen, which must be due to reaction with ascorbic acid with an earlier intermediate in the glucobrassicin breakdown. Early authors (e.g., Bjeldanes, in the 1980s) suggested indoles to be cancer protecting; later authors noted Janus properties, being either protectors or carcinogens depending on the timing of the carcinogen and the indole, as reviewed by, e.g., Holst and Williamson [29] and Agerbirk et al. [26].

Although several studies showed that DIM interferes with diverse signal transduction pathways implied in tumorigenic and inflammatory processes, such as AKT kinase, phosphoinositide 3 kinase (PI3K), NFκB pathway, or EGFR/ERK, the exact interaction has not been yet elucidated [30,31]. Furthermore, it has been studied that the growth and expansion of certain cancer types, as colorectal, are promoted by the presence of pro-inflammatory interleukins, like TNFα and IL-6. These molecules activate pathways regulated by NFκB and transcription factor STAT3, inducing and maintaining in time a pro-inflammatory microenvironment [32]. In this way, carcinogenic cell proliferation, tumor invasion, angiogenesis, and immunosuppression are promoted [33]. The work of Zou et al. [34] showed that when human ovarian cancer cells (SKOV3 and A27809) were treated with DIM, a downregulation in STAT3 and a subsequent inhibition in cell adhesion and invasion was observed.

All these studies performed in vitro suggest that cruciferous vegetables mainly from *Brassica* spp. (either as foods or ingredients rich in bioactive substances) are good candidates as dietary coadjutants to improve human health in NCDs. As a consequence, some clinical and pre-clinical studies have been developed during the last six years. Epidemiological studies have linked the intake of GSLs with the risk of coronary heart disease or type 2 diabetes [35,36]. Nevertheless, this information from large cohorts is usually based in food frequency questionnaires, making it difficult to know the specific effects of the GSLs in the intake. On the other hand, promising anti-cancer results have been obtained from interventional studies but, due to the reduced sample size, cannot be interpreted as a generalization [37]. In general, the studies analyzed in our review did not present an established and standardized protocol, differing in the concentrations of the component, time of the intervention, and sample size. Moreover, not all of them presented a double-blind design. For all that, this review aims to analyze the critical points of the studies performed with *Brassica*-related biomolecules with the aim to establish a basis for future trials and avoid biases. 

## 2. Methodology

In the present work we pursued the objective of critically reviewing the clinical studies in which ingredients and bioactive compounds from cruciferous vegetables (Brassicaceae family) had been used with different pathologies. Based on the anti-cancer properties attributed to GSLs and isothiocyanates (ITCs), we opted to search according to these pathologies since they were the most studied in the past decades. Additionally, one of the main target pathways of the SFN in the organism is the Nrf2–Keap1–ARE system [13,14,15], with a wide role in the development and progression of chronic diseases (e.g., cancers, respiratory problems), and, finally, based on their anti-inflammatory characteristics we opted also to review the more recent research data on the effects of the consumption of GSLs in the development and management of metabolic diseases, mediated by systemic inflammatory conditions. Diverse bibliographical searches were carried out in databases including PubMed, Scopus, and SpringerLink using the following terms and keywords: cancer, metabolic disease, respiratory tract disease, sulforaphane, and glucosinolates. In order to make a more concise selection, the results were limited to clinical trials. From the obtained records, articles including cruciferous foods and derived products were the selected items, and we reduced the timeframe to the last years, from 2012 to the present as well. This selection gave us 53 articles of studies developed with human subjects, with granted access to the full text of the given publication, and contributed with clinical data of the compounds and the pathologies present in the studies. The articles based on in vitro or in vivo (animal models) experiments or trials were not considered because we focused our work on the data of studies carried out with human adults with different pathologies, to back the decision-making when looking for nutritional recommendations in the daily clinical practice with such patients. Nor were articles using low-caloric diets, low-fat diets, or Mediterranean-type diets considered, because these are healthy options for the human subjects in any intervention study, and they did not provide us with specific information about consumption of cruciferous foods or ingredients derived from Brassicaceae products. Additionally, articles where the isothiocyanates (ITCs) were used as biomarkers in intra-hospitalar tumor resections or documents based on healthy volunteers were also not included in the analysis, because they were patients with pathologies unrelated to our objective of study, and therefore, 36 articles were discarded. 

According to all these selected criteria, 15 clinical studies were selected to carry out this critical study of clinical evidence using bioactives from *Brassica* and also one cohort study because of its ample sample size and long period of follow-up of 22 years [35]. We also carried out the analysis of a “letter to editor” [38] that was submitted as a response to this cohort study. 

## 3. The Clinical Evidences of Health-Promotion with *Brassica* Bioactives from a Critical Point of View

In order to proceed with the critical analysis of the articles, they were organized and grouped according time of duration of the study and type of pathology, in short-term or acute studies (Supplementary Table S1), medium duration or length (Supplementary Table S2), and long-term studies (Supplementary Table S3).

### 3.1. Short-Time Studies

Within the short-term studies (Table S1), we found two documents on prostate [39] and melanoma [40], three papers on respiratory pathologies [41,42,43], and one on type 2 diabetes [44]. The main limitation observed in these studies is the length or duration of the study, of 1 month or less, because the validity of the data in terms of changes in bioavailability of the intake of compounds could be acceptable, but for the evaluation of data from tissues, the time of exposition is too low. In the article of Gee et al. [39], a Phase Ib study, studied the concentration of DIM in prostate tissue after its administration in three groups: 200 mg, 400 mg, or placebo. They found a positive increase of DIM in plasma, but not concomitant or related with the presence of DIM in the prostate tissues, and the initial hypothesis was not corroborated. On the other hand, the urinary 2/16-hydroxysterone increased significantly by 68% (*p* = 0.030) in the group administrated with 400 mg of DIM. The levels of PSA taken separately, were also non-significant, but comparing between the group of 400 mg versus placebo, the reduction was of 1.8 times (*p* = 0.1). The gene expression of the CYP1A1 in blood after 15 days in the Group of 200 mg was significantly reduced by 31% (*p* = 0.032) and more importantly for the reduction for the CYP2B6 was highly significant by 87% (*p* = 0.040).

In Tahata et al. [40], patients with more than two nevus or cell-proliferation structures of ≥4 mm Ø were selected and showed significant increases (ratio of concentration >1.5) in 14 out of the 92 proteins identified as tumor suppressors during the time of the study. On the other hand, it was not possible to relate the positive data on the significant reduction of proinflammatory cells such as IP-10, MCP-1, MIG, and MIP-1b to the supplementation of broccoli sprout extract (BSE) enriched in sulforaphane (SFN) because of different problems, such as reduced size in terms of number of participants, samples that were too small, and of a different nevus, which made the analysis difficult and induced higher inter-lesion variability. From the size of the nevus, it was not possible to obtain significant differences, only a general increase in size that was less patent in the patients treated with 100 µg BSE–SFN. Against these results it is worth highlighting that the changes in the size of the nevus in a period of one month are unusual and the comparisons between pictures depends in the illumination characteristic of the images, being unreliable data when analyzing the results. One of the strengths of this work actually are the improvements suggested for the future to use a different technique for the biopsies (in patch), to reduce the stress in the area of lesion, and to use a dermatoscope to substitute the pictures because of their great variability according to the illumination conditions. 

The short-time studies included also the three studies on respiratory problems. In the work of Sudini et al. [41], the first observation was the extremely short time of intervention of three days, the shortest of all the analyzed studies. The intervention was carried out using food, not pills or supplements, because the interest was to study if the intake of cruciferous foods with high content in glucosinolate, especially glucoraphanin (sulforaphane), could have the same effect that was found in the interventions with broccoli sprout extract (BSE). The final objective of this work was to provide evidence to make recommendations of public health to increase the consumption of broccoli sprouts and germinates as a preventive factor in asthma and related pathologies. The study established very clearly the amount of sprouts to be ingested, the method of preparation, the temperature of the rest of foods, the time of chewing the sprouts, as well as the age of the sprouts, in order to reduce the variability in the concentration of glucoraphanin (sulforaphane) ingested. Despite this good design, the very short time of exposure resulted in non-significant results in any of the studied parameters: lung inflammation, oxidative stress, gene expression, antioxidant level, symptoms or lung function. The work of Wise et al. [42], compared to the previous one, increased the time of intervention, but again used a supplement—capsules with different concentrations of SFN. The pathology under study was chronic obstructive pulmonary disease (COPD), and in both cases, asthma and COPD were obstructive conditions. In the exclusion criteria, this one was the only paper that considered that the patients could be under treatment with oral anticoagulants. Other studies mentioned the cardiac pathologies as exclusive and were not as specific with the medication. It is indeed an important exclusion factor, because these drugs are anti-vitamin K and are affected by the dietary intake of green-leafy vegetables and other dietary sources of vitamin K, which may put at risk the correct availability of the drug, affecting the coagulation of blood, and requiring more control of the dosage administrated to the patients. The results obtained, with an statistical potency close to the 80%, were not significant for the expression of the NRF2 factor. The only significant difference in the treated groups was for the AKR1C1, which increased 1.45 times in the placebo versus 1.08 and 0.79, in the groups of 25 µmol and 150 µmol, respectively. Nevertheless, none of these changes was significant with respect to the basal level. The results were not very encouraging, but the authors opened a door to the analysis of the absorption via nasal application of bioactive compounds and its medical implications. Unlike the previous article, Brown et al. [43], found significant results. The strong points of this work worth highlighting are the detailed preparation of the lyophilized material used and the characteristics of the diet description and instructions for the patients specifying the vegetables to be avoided. With respect to the experimental data, the induction with methacholine produced a reduction of 28.7% ± 7.2% in forced expiratory volume FEV1 (measure of lung disease); after consuming the SFN, 60% of the patients with asthma blocked the bronchoconstriction (BC) mediated by methacholine, in 20% of the patients the situation worsened, and in the last 20% there was no response. Besides, a significant reduction in the resistance to the entrance of air was detected (p = 0.03), and a small but significant increase in the average aerial way lumen. The FEV1 results and the NRF2 values were positively correlated between them. The results of bronchoconstriction (BC) and bronchoprotection (BP) were a paradox that needed a multivariable regression model to be understood (R2 = 0.67, *p* = 0.0003), considering the bronchoprotection as a resulting variable and using as independent values the reduction of FEV1 induced by methacholine, and the changes in the genes GCLM, GST1, and NQO1. Using this model, a negative correlation between BP and FEV1, and a positive correlation between BP and NQO1, were found. The authors concluded that SFN increased the expression of the NQO1 gene (phase II enzyme) associated with the increase in FEV1 as a response to the induction of methacholine and the improvement associated independently in the bronchoprotective capacity of deep breathing. The last work in this group of short-time interventions analyzed is about metabolic pathologies, corresponding to the work of Bahadoran et al. [44], with an intervention on 81 patients of type 2 diabetes mellitus within the first year after diagnosis of the disease. A number of participants dropped-out of the study, and at the end, 63 patients (80%) were analyzed for fasting glucose, showing a significant reduction (reduction in both A—225 µmol SFN and B—112 µmol SFN groups), and a significant reduction in the A-group of insulin and insulin resistance. 

### 3.2. Medium-Length Studies

The analysis of the articles of medium-length duration between 2 and 8 months (Table S2) indicated that three were dedicated to studies on cancer in different localizations, two studies were on prostate cancer, and one study was on breast cancer. 

In both studies of Alumkal et al. [45] and Cipolla et al. [46], the main factor studied was the change in the PSA (prostate specific antigen) parameter. In their study, Alumkal et al. ambitiously established the objective of a reduction of 50% in the level of PSA after 20 weeks of intervention, an objective that was not achieved. The obtained reductions were significant and in the range of 3% to 20%. In both studies it was also positive to find an increased time for doubling the amount of PSA after the intervention was found (pre- and post-intervention in placebo from 6.1 to 16.6 months [45]; 9.6 to 31.9 months, in the treated group). 

Cipolla et al. [46] were focused in the changes in PSA over time, which were not significant when adjusting the data to the months (m0, m1, m3, and m6); but, when eliminating the m1, the reduction was of 0.0180 ng/mL/month. Besides, the median of log PSA was 38.5% lower in the intervention group than in the placebo, and the progression of the PSA was by 71.8% in the placebo versus the 44.4% in the SFN-treated group. Looking into the changes of the phase II enzymes, the GSTM1, and the inhibition of the HDAC in the work of Alumkal et al. [45], non-significant differences were found. In both studies, the time for the intervention was similar, namely 20 and 24 weeks, respectively, but the amount of SFN was much higher in the work of Cipolla et al. [46], and that probably favored the better more encouraging final results in this study. In any case, Atwell et al. [47] planned to evaluate the efficacy of the consumption of the broccoli sprouts extract to modify the activity of the HDAC (histone deacetylase) and the breast tumor marker CA (carcinogenic antigen) in order to improve the diagnosis in women with benign disease and non-invasive CA. They proposed an intervention using six pills with a theoretical content of SFN of 30 mg in three administrations per day. A point in favor of this article is that they carried out an internal analysis of the composition of the capsules, and determined the total daily dosage of 224 mg of SFN instead of the declared 180 mg of SFN as designated by the supplier of the supplement. When evaluating the results, the levels of the HDAC before and after administration between groups were not statistically different. Only when comparing the two groups, treated and untreated, after the intervention was a significant difference observed, with a reduction in the HDAC in the group with the treatment. It should be noted that the authors also carried out a stratified data analysis considering the consumption of NSAIDs, using this variable as independent, and they found a significant reduction in the HDAC in the patients that were non-regular consumers of NSAIDs (*p* = 0.04), and it was not possible to find significant differences in the group of consumers of NSAIDs. Additionally, the biomarkers H3K18ac, H3K9ac, HDAC3, HDAC6, Ki-67, and P21 did not present statistically significant differences in the pre- or post-analysis. When comparing the levels of pre- and post-treatment within each group, a significant reduction was found in Ki-67 and HDAC3 in benign tissues in the SFN treated group, and a significant reduction of H3K9ac in the tissular DCIS in the placebo group. As a conclusion of this work, the authors did not plan as a hypothesis that the NSAIDs inhibited the synthesis of prostaglandins, and this could suppress the expression of regulator proteins through the involvement of the recruitment of the HDAC. The increased recruitment of the HDAC in the chromatin could prevent the inhibition associated with the consumption of SFN, and that could be a potential explanation for the observed data, and for the future studies using interventions with SFN, longer studies will be required to confirm these findings. 

### 3.3. Long-Term Studies

Finally, we proceeded with the analysis of the articles studying long-term interventions (Table S3). In these works, we found various publications on different types of cancers: prostate [48,49], breast [37], ovarian [50], and pancreas (pilot [51] and development [52]), and one work on type 2 diabetes mellitus [35] and a letter to the editor [38] as a result of this study. 

The works of Paltsev et al. [48] and Traka et al. [49] were both dedicated to prostate cancer, for a period of 12 months, but with different interventions and different parameters of the stage of development. In the first one [48], the intervention was carried out using capsules with 900 mg of DIM (3,3-diindolylmethane) and considered data of morphological changes (MI formula to calculate presence of cancer) in the prostate, and registration of the prostatic intraepithelial neoplasias (PIN) of low and high degree as well as the number of foci of cancer and urological dynamic parameters (maximum, average, and residual urinary flow) and erectile function. In the second work [49], with a higher dosage of glucoraphanin (parental glucosinolate of SFN) through the intake of different broccoli soups (based on broccolis with different contents of glucoraphanin), specific parameters included the change in the genetic expression of the RNA, changes in metabolites after biopsy from prostate tissue, and the plasma PSA levels. In the results of Paltsev et al. [48], a significant reduction in the cancerous and precancerous cells was obtained in the treated group (from MI = 50 before treatment to MI = 0.08 after treatment), obtaining a complete regression in 45.5% of the patients (5 out of 11). Despite the results, the extremely reduced sample size in this study made it impossible to establish a significant difference between groups. On the other hand, Traka et al. [49] observed changes in the genetic expression in all the patients, considering as reference the group that took the soup with the unmodified broccoli (not enriched). The changes were more striking in the patients that took the soups with enriched broccoli (with much higher glucoraphanin than regular broccoli, through the presence of one or two alleles of the Myb28 gene), but the study did not reach a significant result, because the number of patients or participants was below the minimum necessary of 78 participants, in order to detect significant changes of *p* < 0.02. The authors concluded that a diet rich in glucoraphanin may attenuate the transcriptional changes that take place in the prostate, but the data were not conclusive because of that limitation. It became clear, as in other studies with natural products and interventions for different types of diseases, that the size and number of participants should be enough to generate sound results that would help in the elaboration of recommendations for dietary options with food rich in bioactives such as *Brassica* vegetables, microgreens, and sprouts. 

The works of Thomson et al. [37] on breast cancer and Kiselev et al. [50] on ovarian cancer were studies with the highest number of participants and the longest periods of evaluation. Thomson et al. [37] studied the effects of diindolylmethane (DIM) + tamoxifen administration for 12 months. Previously, a tolerance test was carried out with the 10 first participants to establish a 150 mg dosage of DIM. From the aims of the study, the main objective was to analyze the breast density, and it had to be discarded because of the high incidence of participants with bilateral mastectomy, and therefore, they analyzed the effects on 2-OHE/16-α-OHE (alpha hydroxylated estrogen), the levels of strone, stradiol, and SHBG (sex hormone binding globulin). The statistical power of this study was established under 88% because it needed at least 55 participants per group, and in this case, the study was finished by 51 patients in the placebo group and 47 in the intervention group. The results support the hypothesis that the DIM (from *Brassica*) induced an increase in the 2-OHE/16-α-OHE in urine (*p* = 0.001) and increased the circulating SHGB in the patients with the combined treatment with tamoxifen. Besides that, the pharmacokinetic of the tamoxifen was changed, reducing the circulating forms of this drug. The results are positive but cannot be used for generalization. 

On the other hand, the work of Kiselev et al. [50] provided data on the survival and the survival without progression of ovarian cancer at 5 years in the patients with the combined treatment of EGCG (epigallocatechin gallate of green tea) and indole-3-carbinol (I3C)—the precursor of DIM. The combined I3C + EGCG demonstrated a 1.5 times increase in the survival free of cancer, a significant reduction in recurrences of the ovarian cancer with ascites in the treated group (by 8%–9%) when compared with the untreated group (by 60%–63%). The first results were observed in the pre-surgery stage because the majority of the patients (81%–85%) in the therapeutic branches of maintenance 1–3 could take a successful reduction surgery to eliminate all the visible tumor focus. On the contrary, for the participants in branches 4–5, it was not possible to carry out such procedure. Additionally, the levels of CA-125 were statistically lower in the 1–3 pre-surgery groups and after treatment than in the control group. Based on these data, the authors concluded that these were preliminary results, but they hypothesized that the treatment of I3C + EGCG, administered as part of the maintenance therapy during and after the combined treatment, inhibited the cancer precursor cells at the ovarian level, and therefore, diminished significantly the incidence of recurrence (ascites), and this was also redundant for higher rate of survival, global survival, and free-of-cancer survival. 

From the article of Lozanovski et al. [51], a pilot study, we found that one of their inclusion criteria made it difficult to extrapolate the results to all the participants, because it only considered patients without gastrointestinal symptoms, which are common symptoms of this pathology and in the chemotherapy treatments. With respect to the objective of the study, they aimed to analyze the effects of the intake of encapsulated broccoli sprouts as co-adjuvants in the chemotherapy and proposed the intake of 15 capsules of broccoli sprouts at a time, giving 90 mg of SFN and 180 mg of glucoraphanin, a never before tested dosage, which was established because of the poor life expectancy of the patients and the aggressive characteristics of this type of cancer. The results of Lozanovski et al. [52], later on, established that the secondary effects of the chemotherapy, the lack of appetite, nausea, vomits, diarrhea, mouth sores, etc., were factors that made it very difficult for the patients to intake 15 pills at once, as the study initially planned. Besides, the masking of the bioactive agent was not possible because the pills were easily distinguishable. All these raised inconveniences, the progression of the pancreatic cancer and the GI symptomatology, led to a high rate of drop-off of 72% in the treatment group and 55% abandonment in the control group, and therefore, the results were not significant. 

In order to finish this critical analysis, the only study of cohorts included in this review, from the group of Ma et al. [35], analyzed data from 200,907 people free of disease (cancer, diabetes mellitus, or cardiovascular disease) at the beginning of the study. To establish this, they used semi-quantitative polls of food frequency: vegetables, fruits, whole grains, sugar drinks, juices, nuts, legumes, red meat, processed foods, trans-fats, omega-3 fatty acid rich foods, polyunsaturated fats, salt, and alcohol consumption. They also included the risk factors for type 2 diabetes mellitus (T2DM), body weight, smoking, pharmaceutical treatments or vitamin complex intake, family history of diabetes, chronic diseases, hypertension, dyslipidemia, and physical activity calculated on a weekly basis. With all this information, the authors presented the results that there was a risk for T2DM with the intake of cruciferous foods and glucosinolates. However, in agreement with the work of Oliviero et al. [38], this study presented big limitations because it did not review or consider the cooking procedures, it did not quantify the amount of vegetables consumed on a daily or weekly basis, and it did not quantify the glucosinolate contents in the different vegetables consumed. Besides, the population sample was homogenous, because all of the participants were health-area workers, and the results are not extrapolatable to the general population. Finally, it should be highlighted that other nutrients and cofounding factors were not considered in the study, and they could affect the development of these pathologies. 

## 4. Future Perspectives and Challenges

The assessment of the health effects of bioactive compounds from plant-derived foods requires several aspects to be taken into consideration and a different perspective from that when evaluating single compounds of any chemical entity. In the case of *Brassica* spp. and other cruciferous foods and bioactive compounds obtained from them, it is crucial to control the concentration of glucosinolates ingested or delivered in the administration, and therefore to control the factors that affect this composition in the natural matrix, namely the production practices, handling, cooking procedures, time of chewing, age of sprout/mature plant, amount ingested, and concomitant ingestion with other foods, etc. In this context, well-researched studies are being carried out that include and analyze all these factors and standardize the *Brassica*-derived products by their SFN content [53,54], and efforts must continue in this line. The exact calculation of the amount of raw broccoli needed to produce a supplement that delivers a specific amount of SFN and its metabolites within the body remains to be established [55]. It is important to point out that a food product should be tested in its pattern of consumption on a daily basis or with a frequency of consumption clearly reported.

One point that deserves attention is the unreasonable but common focus in the literature on glucoraphanin and glucobrassicin that merits mention, and contrasted to the high number of other GSLs occurring in vegetables. There are evidences presented for exciting protective effects of many other GSLs, but they are not commercially available [56,57]. The same could be said for the indoles, with the literature focusing to an absurd degree on compounds that happen to be commercially available, although they are not necessarily the most widespread in vegetables [58], or when comparing a non-substituted and a substituted indole [59].

Similarly, efforts on health-oriented breeding (e.g., manipulation of the activity of nitrile specifier protein in broccoli by Roman et al. [60]) as well as the very promising bioactivities of exotic GSLs like glucomoringin and its ITC moringin from a tropical tree not from the Brassicaceae family but within the order Brassicales [57]. This area of work together with additional efforts on the discovery of brassicaceous phytochemicals is another perspective: the widespread occurrence of epithionitriles in cabbage and new GSL products in these plants [10]; the poorly known chemistry of some vegetables, e.g., commercial chemotype of watercress in the USA [61]; and other derivatives including seleno-glucosinolates [62,63].

Additionally, the potential for positive synergistic effects of combinations of GSLs have been little studied, even though some pioneering work can be found in this subject [64]. These are all examples of the many open lines of work for the future of understanding the potential benefits of the consumption of the bioactive compounds present in cruciferous foods from the farm to the foods for health.

The CONSORT (Consolidated Standards of Reporting Trials) statement includes a check list as a guidance for reporting randomized controlled trials and hence, it serves indirectly as a guidance for the design of human intervention studies to evaluate the beneficial effects of foods [65]. The report of the trial design must include the eligibility criteria and a participant flow diagram, complete data of the intervention administered, and well-defined primary and secondary outcome measures, among others. It is highly advisable to refer to this check list before performing an intervention to ensure that we take into account all the factors that may later influence the final result.

In human studies, sample size is an important factor to reduce inter-individual variability, especially in markers subjected to a great variability, as the expression of particular genes, but for logistical reasons it is not always possible to have a large and appropriate number of participants. However, to help to reduce the variability in clinical trials performed in parallel groups, it is essential to control variables (the so-called covariables) that can influence in the behavior of other variables. Sex, age, and BMI are covariables that greatly influence responses linked to inflammatory processes or gene expression. It is important to randomize intervention groups stratified by these covariables, in order to reduce inter-individual variability, so that significant differences are more likely to be detected, and some clinical trials do not take these aspects into account. Absorption, distribution, metabolism, and excretion (ADME) processes are also influenced by age, sex, dietary habits, or the microbiome. The COST Action POSITIVe working group has recently published the determinants involved in the ADME process of different bioactive compounds [66], and they include the concept of metabolic phenotype (metabotype) or the similar pharmacokinetics profile common in some individuals, which may result in similar response patterns. This concept should be applied to the human host as well as to the gut microbiota metabolisms and is dependent on gene polymorphisms as well as microbiome composition. This perspective can help to understand the differences in responses observed between individuals. Stratification of individuals by metabotype is an attractive challenge for future research. 

It is crucial that the outcome measure is of biological relevance; biomarkers or risk factors measured must be associated to the latter development of a disease. Validation of appropriate markers is a pending issue as very few are recognized as valid in terms of specificity and applicability to a range of population [66,67].

Biomarkers of effect are early stage end-points, for instance the modulation of phase 2 enzymes by glucosinolates. They need more time than biomarkers of exposure to be influenced by the dietary treatment. Hence, length or duration of the study must be defined according to the biomarker measured to be modified, that is, to define perfectly the time of exposure to observe changes in relevant parameters. Gene expression is one important target for glucosinolates, and it requires a sufficient period of exposure to (de)activate signaling pathways involved. It is crucial to find appropriate biomarkers of effect that are linked to later disease outcomes, and more investigation is needed in this sense. Post-study follow-up can be of great value in assessing the persistence of certain effects, or in discovering those that appear more long-term [67]. 

In case of clinical trials performed with patients and not healthy subjects, the appropriate timing of treatment with the nutraceutical or food product is also a pending issue. The degree of progression of the disease greatly influences the response observed. In some cases, a bioactive compound is no longer sufficient, and in other cases it can have the opposite effect. It has been suggested that the effect of SFN on Nrf2 activation is desirable in the early stages of tumorigenesis, whilst it may have a deleterious effect in later stages. It can behave as an oncogenic transcription factor and increase the cytoprotective activity in the cancer cell and cause resistance to chemotherapy [67]. More studies are needed on the potential effects of this molecule on patients with diagnosed cancer before making any recommendations of supplements with these phytochemicals [55]. The need for studies with larger populations is clear, as well as longer follow-ups, in order to find significant results with enough strength to support recommendations for public health in favor of increasing the consumption of cruciferous foods on a daily basis. 

## Supplementary Materials

The following are available online. Table S1: Short-time studies; Table S2: Medium-length studies; Table S3: Long term studies.
