# Supplementary material for: The Role of Brassica Bioactives on Human Health: Are We Studying It the Right Way?
Source: Molecules. 2020 Mar 30;25(7):1591. doi: 10.3390/molecules25071591 (PMC7180841; doi:10.3390/molecules25071591)
Supplement: Supplementary file 1 [file molecules-25-01591-s001.pdf]

Supplementary Table S1. Short-time studies

Reference	Pathology	Type of Study	Sample size	Time of evaluation (days)	Type of intervention	Variables under study
(39)	Prostate cancer	Randomized clinical study	N=45	21-28	DIM in dry formula 3 intervention groups (200 mg, 400mg and placebo)<br>-	Plasma and prostate DIM levels<br>Urinary 2OHE/16OHE.<br>PSA and testosterone level<br>IGF-1:IGFBP-3 ratio.<br>mRNA in CYPs
(40)	Atypical Melanoma	Stratified and randomized clinical study	N=17	28	1 gel capsule of BSE-SFN.<br>3 groups: 50, 100 and 200 µmols of BSE-SFN	Pictures, biopsia, SFN in skin and plasma, Plasma citokines
(41)	Bronchospasm and airway inflammation	Double-blind placebo-control randomized clinical study	N=40	3	Broccoli sprouts (BS)<br>2 groups: 100 gr BS rich in SFN; 100gr alfalfa	FENO<br>Lung Function Test<br>Nasal epithelial cells, TSH values in urin and blood
(42)	COPD	Double-blind multicenter randomized clnical study with control group of three arms for 2 concentrations of SFN.	N= 89	30	SFN capsules (BSE)<br>3 groups:<br>- BSE 4.4mg (25µmol SFN)<br>- BSE 26.6mg (150 µmol SFN)<br>- Placebo: microcelulose	NRF2 in macrophages and bronquilal epithelium cells.<br>Concentration of antioxidants.<br>Inflammatory markers. Lung function and symptoms of the patients.
(43)	Patients with Asthma	One arm clinical study without control group	N=51, selected.<br>N=44 with data	14	1 group before/after<br>20 mL bottels of mango juice + 100 µmol of SFN disolved; frozen, and ingested once a day in the afternoon.	Bronchoconstriction<br>Reduction of FEV1<br>Nfr2 factor activity
(44)	Patients with T2DM	Double-blind randomized clinical study with control group	N=80<br>(N=63 at the end of the study)	28	Broccoli sprouts (BS):<br>- 10 g/d BS: 225 µmol SFN<br>- 5g/d BS : 112 µmol SFN<br>- Placebo:corn flour with chlorophyll	Fasting glucose levels<br>Insulin resistance<br>Serum insulin

**Supplementary Table S2.** Medium-lenght studies						
Reference	Pathology	Type of Study	Sample size	Time of evaluation (weeks)	Type of intervention	Variables under study
(45)	Patients with recurrent CA and PSA (prostate)	One arm clinical study without control group.	Initial N= 20<br>N= 16 finished the study.<br>N = 2 eliminated (Elevated PSA)<br>N= 2, dropped-out, GI secondary effects or did not follow the visits.	20	1 group<br>Capsules with 218 mg of SFN from Broccoli sprouts (200 µmol of SFN, 4 capsules, before breakfast, once a day)	Reduction of 50% of PSA levels after the 20 weeks.<br>Percentage change in PSA.<br>Changes in GSTM1.<br>Inhibition of HDAC<br>Time for double the PSA
(46)	CA in prostate after radical prostatectomy	Double-blind placebo-control randomized clinical study	N=78 (6 months)<br>N=75 (8 months)	32	Dosages of 10 mg SFN pills.<br>2 groups:<br>- 60 mg/d (2 pills/3 times/d x 24 weeks + 8 weeks of follow-up)<br>- Placebo: Same pills, no treatment.	Reduction of PSA.<br>Time to duplicate PSA.<br>Progression of PSA.<br>Differences in the adverse effects in the groups.
(47)	Non-invasive Breast cancer	Double-blind placebo-control randomized clinical study	N= 54	8	SFN capsules<br>2 groups:<br>- 2 capsules/3 times/d (224 mg SFN/d)<br>- Placebo: microcristaline cellulose	Polls: family history and risk factors; food questionaires.<br>Concentration of SFN metabolites in blood and urine.<br>Blood mononucleus cells. Histone deacetylase activity.<br>Markers: H3K18ac, H3K9ac, HDAC3, HDAC6, Ki-67, p21

**Supplementary Table S3.** Long-term studies						
Reference	Pathology	Type of Study	Sample size	Time of evaluation (Years)	Type of intervention	Variables under study
(48)	CA prostate	Double-blind randomized clinical study with placebo	N= 21	1	2 groups<br>- Infemin: 900mg DIM/d (3 capsules)<br>- Placebo	Morfological changes in prostate<br>Urodynamic parameters<br>GI symptoms<br>Erectyl disfunctions
(49)	CA prostate	Clinical study upon pilot study (with healthy patient). Double-blind randomized clinical study.	N= 61	1	3 Groups (Glucoraphanin – parental of SFN):<br>- X soup: Myb28 B/B broccoli genotype<br>- Y soup, Myb28 B/V (Myb28 allele introgressed from *B. villosa*)<br>- Z soup, Myb28V /V	Changes in genetic expression (RNA)<br>Metabolites in the biopsia of prostates<br>Blucose, PSA and Lipids in blood extraction
(37)	Breast cancer	Double-blind randomized clinical study with placeb	N= 130	1	Diindolylmethane (DIM)+ tamoxifen<br>2 groups:<br>- 150 mg DIM/12 h<br>- Placebo	2OHE1, and 16αOHE1 levels and ratio<br>SHBG<br>Breast density
(50)	Ovarian cancer	Clinical study. Free election of treatment by the participant patients.	N= 284	5	Indole-3-carbinol (I3C) in combination with anticancer therapy (AT):<br>- **N=** 46; AT + 2 (oral 200 mg I3C), continued administration<br>- **N=** 76; AT + oral 200 mg I3C + 200 mg EGCG, twice daily, continued administration<br>- **N:** 42 AT + 2(200 mg I3C+ 200 mg EGCG), continued administration and long term chemotherapy (Platinum-taxane, 2-3 month periods)<br>- **N:** 40, combined treatment without chemotherapy<br>- N=80 combined therapy	Survival and survival free of cancer<br>CA-125, and PET-CT<br>MRI<br>Ultrasound graphics
(51)	Pancreatic cancer (terminal)	Monocentric Pilot study	N= 40	1	15 capsules of freeze-dried broccoli sprouts, once a day (90 mg SFN = 15 capsules of 400 mg of broccoli sprout powder , 6 mg each). And treatment with active chemotherapy.	Serum CA19-9 and CEA<br>Image tests and Uring ITCs<br>Quality of life (validated scales)<br>Food questionaires,

(52)	Pancreatic cancer (terminal)	Randomized Clinical study with control group	N= 40	1	15 capsules of freeze-dried broccoli sprouts, once a day (90 mg SFN = 15 capsules of 400 mg of broccoli sprout powder , 6 mg each).<br>Treatment with active chemotherapy.	Therapeutic follow-up
(35)	Type 2 Diabetes Mellitus (T2DM)	Cohorts, prospective, patients free of cancer, T2DM or cardiovascular disease	N=200,907<br><br>(4,303,750 people-year)	22	Free living people with no specific intervention<br>3 cohorts<br>Food questionaires (including the cruciferous foods)	Food frequency questionaires, 9 categories, and registration of different habits:<br>Alcohol consumption; risk factors for T2DM, body weight, smoking, physical activity, prescription drugs, vitamin intake, family history of diabetes, chronic diseases, and physical activity evaluation on a weekly basis.
